# Endothelial biomarkers (Von willebrand factor, BDCA3, urokinase) as predictors of mortality in COVID-19 patients: cohort study

**DOI:** 10.1186/s12890-024-03136-0

**Published:** 2024-07-04

**Authors:** Rocío Nayeli Sánchez-Santillán, Martha Patricia Sierra-Vargas, Dulce González-Islas, Octavio Gamaliel Aztatzi-Aguilar, Rogelio Pérez-Padilla, Arturo Orea-Tejeda, Yazmín Debray-García, Manolo Ortega-Romero, Candace Keirns-Davis, Alejandra Loaeza-Roman, Alejandra Rios-Pereda

**Affiliations:** 1 Heart Failure and Respiratory Distress Clinic, Cardiology Service, Ciudad de México, Mexico; 2 Subdivision of Clinical Research, Instituto Nacional de Enfermedades Respiratorias “Ismael Cosío Villegas”, Ciudad de México, 14080 Mexico; 3 Department of Toxicology, CINVESTAV-IPN, Ciudad de México, 07360 Mexico; 4 Department of Research on Tobacco and COPD, Instituto Nacional de Enfermedades Respiratorias “Ismael Cosío Villegas”, Ciudad de México, 14080 Mexico; 5 Department of Toxicology and Environmental Medicine Research, Instituto Nacional de Enfermedades Respiratorias “Ismael Cosío Villegas”, Ciudad de México, 14080 Mexico; 6 Cardiology Department, Instituto Nacional de Enfermedades Respiratorias “Ismael Cosío Villegas”, Calzada de Tlalpan, 4502 Col Sec XVI, Del Tlalpan CP 14080 , Mexico City, Mexico

**Keywords:** COVID-19, Endothelial dysfunction, Mortality, Von Willebrand factor, SARS-CoV-2, uPA, BDCA3

## Abstract

**Background:**

SARS-CoV-2 is a systemic disease that affects endothelial function and leads to coagulation disorders, increasing the risk of mortality. Blood levels of endothelial biomarkers such as Von Willebrand Factor (VWF), Thrombomodulin or Blood Dendritic Cell Antigen-3 (BDCA3), and uUokinase (uPA) increase in patients with severe disease and can be prognostic indicators for mortality. Therefore, the aim of this study was to determine the effect of VWF, BDCA3, and uPA levels on mortality.

**Methods:**

From May 2020 to January 2021, we studied a prospective cohort of hospitalized adult patients with polymerase chain reaction (PCR)-confirmed COVID-19 with a SaO2 ≤ 93% and a PaO_2_/FiO_2_ ratio < 300. In-hospital survival was evaluated from admission to death or to a maximum of 60 days of follow-up with Kaplan-Meier survival curves and Cox proportional hazard models as independent predictor measures of endothelial dysfunction.

**Results:**

We recruited a total of 165 subjects (73% men) with a median age of 57.3 ± 12.9 years. The most common comorbidities were obesity (39.7%), hypertension (35.4%) and diabetes (30.3%). Endothelial biomarkers were increased in non-survivors compared to survivors. According to the multivariate Cox proportional hazard model, those with an elevated VWF concentration ≥ 4870 pg/ml had a hazard ratio (HR) of 4.06 (95% CI: 1.32–12.5) compared to those with a lower VWF concentration adjusted for age, cerebrovascular events, enoxaparin dose, lactate dehydrogenase (LDH) level, and bilirubin level. uPA and BDCA3 also increased mortality in patients with levels ≥ 460 pg/ml and ≥ 3600 pg/ml, respectively.

**Conclusion:**

The risk of mortality in those with elevated levels of endothelial biomarkers was observable in this study.

**Supplementary Information:**

The online version contains supplementary material available at 10.1186/s12890-024-03136-0.

## Background

In December 2019 a new disease emerged in Wuhan, China. In March 2019, the World Health Organization declared the coronavirus disease (COVID-19) outbreak caused by severe acute respiratory syndrome coronavirus 2 (SARS-CoV-2) a pandemic [1]. Immunosuppression, diabetes, and malignancy are associated with severe COVID-19, while older age (> 65 years), male sex, diabetes, and hypertension are associated with increased mortality [2].

One of the main characteristics of SARS-CoV-2 is its ability to spread inside the body and activate endothelial cells (ECs), leading to coagulation disorders such as disseminated intravascular coagulation (DIC), microthrombi, microvascular thrombosis, venous thromboembolic disease and stroke [3–6]. All these conditions are associated with high mortality [7–9].

The endothelium consists of a monolayer of ECs that form the intima, which lies on the inner layer of blood vessels and is protected by pericytes [10, 11]. In the pulmonary system, pulmonary ECs are the basic barriers between the blood and interstitium; they represent one-third of the cells in the lung [11, 12]. The endothelium functions as a protective barrier but also regulates vessel tone and participates in platelet activation, leukocyte adhesion, fibrinolysis, thrombosis, inflammation, and overall homeostasis [13].

The activation of the coagulation pathway, possibly accompanied by DIC, is one of the characteristics of severe infection; an increase in fibrin degradation fragments (D-dimer) is one of the common findings in patients with severe COVID-19 [6]. SARS-CoV-2 enters the pulmonary circulation via the alveoli. First, the inflammatory pathway is activated, accompanied by neutrophil infiltration, extracellular neutrophil trap (NET) formation and thrombus formation [6, 14]. Once the endothelium is activated by interleukin 1β and tumor necrosis factor α (TNF-α), the coagulation pathway starts by displaying P-selectin, von Willebrand factor (VWF), fibrinogen, and platelets. Moreover, ECs release trophic cytokines, which increase platelet overproduction and release vascular endothelial growth factor (VEGF); VEGF interacts with ECs, increasing the expression of tissular factor, which is the main activator of the coagulation cascade [15].

Blood dendritic cell antigen-3 (BDCA3) is a cell surface-expressed transmembrane glycoprotein also known as thrombomodulin. Its presence indicates endotheliopathy and platelet activation, which are factors important in the physiopathology of COVID-19 coagulopathy. Urokinase PA (uPA) is a potent activator of plasminogen and thus fibrinolysis, involving inflammatory processes and endothelial cell migration. It also seems to play an important role in VEGF-induced vascular permeability changes [16–18].

Several markers of endothelial dysfunction are increased in patients with severe COVID-19, and some of these markers are associated with in-hospital mortality [8, 19]. Nevertheless, there is not enough evidence about the roles of VWF, BDCA3, and uPA in mortality. Therefore, the main purpose of this study was to determine the effect of high levels of endothelial biomarkers on mortality.

## Methods

### Ethics

The study was conducted in accordance with the Declaration of Helsinki and its later amendmants. The protocol was approved by the ethics committee of the Instituto Nacional de Enfermedades Respiratorias “Ismael Cosío Villegas” (approval number E06-20). According to the general law of Health Article 17, the study is classified as not at risk. All patients´ data were taken anonymously from their medical records and no identification data was presented. Informed Consent Statement: Patient consent was waived, as it was not required by the ethics committee (approval number E06-20) due to the observational nature of the study, and the research does not involve ethical concerns.

We prospectively studied a cohort of patients admitted to Instituto Nacional de Enfermedades Respiratorias “Ismael Cosío Villegas” for COVID-19 from May 1, 2020 to January 31, 2021. The data came from electronic records, and blood tests were obtained as part of routine evaluation. ECs were obtained from routine blood samples taken during hospitalization during the first hour of the day. All the recruited hospitalized patients were older than 18 years and had a positive RT-PCR result for SARS-CoV-2 in a nasopharyngeal swab, with clinical signs compatible with COVID-19, an oxygen saturation ≤ 93%, and a PaO_2_/FiO_2_ ratio < 300.

Clinical data were collected from electronic records from the emergency room, and laboratory findings were obtained during hospitalization. Patients with HIV, shock, or multiple organ failure (defined as two or more failing systems in the emergency room) at onset were excluded. All patients were treated according to the protocol of the institute and for research no identifying information was presented.

Demographic (age, sex, height, and weight) and clinical variables (comorbidities, mechanical ventilation, treatment, and intrahospital stay) were recorded (Table S1). General laboratory and endothelial biomarker data were collected from the first to the fourth day after hospital admission. Blood plasma was centrifuged to separate the plasma fraction, which was frozen at -80 °C until the biomarkers were measured. All the samples were thawed and immediately analyzed for a total of one freeze-thaw cycle before use.

### Measurement of endothelial markers

Human E-selectin/CD62E and endothelin-1 levels were measured using a human enzyme-linked immunosorbent assay (ELISA) kit (Cat. SSLE00 and Cat. SET100 R&D Systems, Inc. Minneapolis, MN, USA, respectively). Leukemia inhibitory factor (LIF), renin, serpin A4/kallistatin, thrombomodulin/BDCA-3, Urokinase-type plasminogen activator (uPA)/, uromodulin, and VWF were used with the Luminex Human Discovery Assay (7-Plex Cat. No. LXSAHM-07, R&D Systems, Inc. Minneapolis, MN, USA). The measurements were performed following the specifications of each kit. The metabolites reported in this study are expressed in pg/mL.

### Statistics

For the sample size and power calculation, we used the G*power statistical power analyses [20]. The data used for the calculation came from the study of Falcinelli [15], giving us a total sample size of 157 patients.

Variables were evaluated for normality with the Shapiro-Wilk test. Comparisons among study groups were analyzed with chi-square tests or Fisher’s F tests for categorical variables and independent Student’s t-tests or Mann-Whitney U tests for continuous variables. In-hospital survival was evaluated from admission to death or to a maximum of 60 days of follow-up with Kaplan-Meier survival curves and Cox proportional hazard models as independent predictor measures of endothelial dysfunction, including VWF, BDCA3, and uPA, dichotomized by the median cutoff point of our sample. For this study, each EC variable was evaluated in different models. VWF was taken as a principal factor of study and is shown in Table 2. uPA and BDCA3 are shown in the Kaplan-Meier plots.

We ran three models: one crude and two adjusted. Variables with *p* < 0.10 were selected for adjustment of each model. Model 1 was adjusted for cardiovascular diseases (CVD) incidence, doses of enoxaparin, lactate dehydrogenase, direct and indirect bilirubin, and age. Model 2 was adjusted for CVD incidence, dose of enoxaparin, lactate dehydrogenase, total bilirubin, procalcitonin, and age. The likelihood ratio test was used to compare both models. The best model was selected. The statistical analysis was performed using STATA version 14 (Stata Corp., College Station, TX, USA).

## Results

A total of 165 patients (73% men) were admitted to the study, with a median age of 57.3 ± 12.9 years. The most common comorbidities were obesity (39.7%), diabetes (30.3%), and systemic arterial hypertension (SAH) (35.4%) (Table 1).

**Table 1:** Baseline characteristics

Variable	All (*n* = 165)	Survivors (*n* = 135)	Nonsurvivors (*n* = 30)	*p*
Age (years)	57.3 ± 12.9	56.3 ± 1.10	61.4 ± 2.40	0.051
Weight (kg)	80.7 ± 17.9	81.2 ± 17.4	78.1 ± 20.1	0.429
Height (m)	1.63 ± 0.09	1.63 ± 0.09	1.63 ± 0.09	0.970
BMI (kg/m^2^)	30.2 ± 6.20	30.5 ± 6.3	29.0 ± 5.6	0.282
Sex (%) Male	121 (73.3)	98 (73.1)	23 (74.2)	0.904
Smoking (%)	43 (31.4)	34 (30.9)	9 (33.3)	0.808
**Medical history**				
Obesity (%)	56 (39.7)	47 (41.6)	9 (32.1)	0.360
Classification (%)				
I	31 (55.4)	27 (57.5)	4 (44.4)	
II	15 (26.8)	12 (25.5)	3 (33.3)	0.690
III	10(17.9)	8(17)	2 (22.2)	
DM (%)	43 (30.3)	31 (27.4)	12 (41.4)	0.145
SAH (%)	51 (35.4)	42 (36.5)	9 (31.0)	0.581
COPD (%)	3 (2.1)	2 (1.8)	1 (3.5)	0.496
Nephropathy (%)	4 (2.8)	2 (1.7)	2 (6.9)	0.185
CVD (%)	1 (0.7)	0	1 (3.5)	0.203
PH (%)	3 (2.1)	2 (1.7)	1 (3.5)	0.496
**Laboratory findings**				
Glucose (mg/dl)	144 (108–196)	137 (104–180)	171.5 (132–212)	0.032
Total Proteins (gr/dl)	6.9 ± 7.2	7.0 ± 8.1	6.2 ± 0.7	0.271
Albumin (gr/dl)	3.2 ± 0.57	3.2 ± 0.61	3.1 ± 0.44	0.555
Leucocytes (mg/dl)	12.34 ± 5.11	12.23 ± 5.08	12.81 ± 5.27	0.590
Neutrophils (mg/dl)	13.44 ± 14.3	13.29 ± 14.11	14.10 ± 15.84	0.567
Lymphocytes (mg/dl)	0.8 (0.5–1.2)	0.8 (0.5–1.2)	0.65 (0.4-1.1)	0.127
Neutrophil/lymphocyte ratio	9.08 (5.19–18.09)	8.4 (4.53–15.28)	15.88 (10-30.33)	< 0.001
Total Bilirubin (mg/dl)	0.72 ± 0.47	0.69 ± 0.42	0.83 ± 0.65	0.285
Direct Bilirubin (mg/dl)	0.24 ± 0.24	0.22 ± 0.17	0.31 ± 0.42	0.254
Indirect Bilirubin (mg/dl)	0.44 ± 0.22	0.43 ± 0.21	0.50 ± 0.28	0.175
D-dimer (µ/ml)	1.14 (0.56–2.43)	1.16 (0.56–2.48)	0.86(0.6–2.38)	0.839
Pro calcitonin (ng/ml)	0.35(0.15–0.91)	0.32 (0.14–0.89)	0.52(0.26–1.06)	0.066
Fibrinogen (mg/dl)	723.5 (611–874)	714(611–871)	736(639–903)	0.838
C-Reactive Protein (CRP) (mg/dl)	20.4 ± 10.5	19.8 ± 10.8	22.8 ± 9.2	0.169
Troponin (pg/ml)	11.9 (4-74.8)	11.1 (3.7–42.7)	26.6 (4.6-253.8)	0.050
Brain natriuretic peptide (BNP) (pg/ml)	58.5 (27.1-132.5)	55.7 (25.2–118)	70.8 (37.3-384.3)	0.172
**Endothelial function biomarkers (pg/ml)**				
VWF	4899.9 ± 1844.9	4884.2 ± 1905.4	4965.3 ± 1596.1	0.832
BDCA3	3600.1 (2160.5-7239.7)	3300.6 (1913.5-5857.3)	6800.7(3146.3-13467.4)	0.001
Renin	1278.7 (815.5-2293.6)	1278.7 (682.4-2231.9)	1385.7(939.10-3298.7)	0.170
uPA	459.7 (272.1-674.5)	410.8 (260.3-662.8)	593.3 (405.4-756.4)	0.025

COPD: Chronic Obstructive Pulmonary Disease; CVD: Cerebrovascular Disease; DM: Diabetes Mellitus; DVT: Deep Vein Thrombosis; OSA: Obstructive Sleep Apnea; PH: Pulmonary hypertension; SAH: Systemic Arterial Hypertension. Chi square or F fisher test was done for qualitative and independent variables; Student’s t or Mann‒Whitney U for quantitative variables

At the time of hospitalization, the patients had a mean of 9.9 ± 5.7 days of symptoms, and the mean duration of hospital stay was 30.5 ± 18.2 days (Table S1). The sample was divided into two groups according to mortality. A total of 135 (81.8%) subjects survived at 60 days of follow-up, and the remaining 30 (18.2%) subjects died during hospitalization, with 6.2 deaths per 1000 person-days (95% CI: 4.35–9.02).

**Fig. 1:**
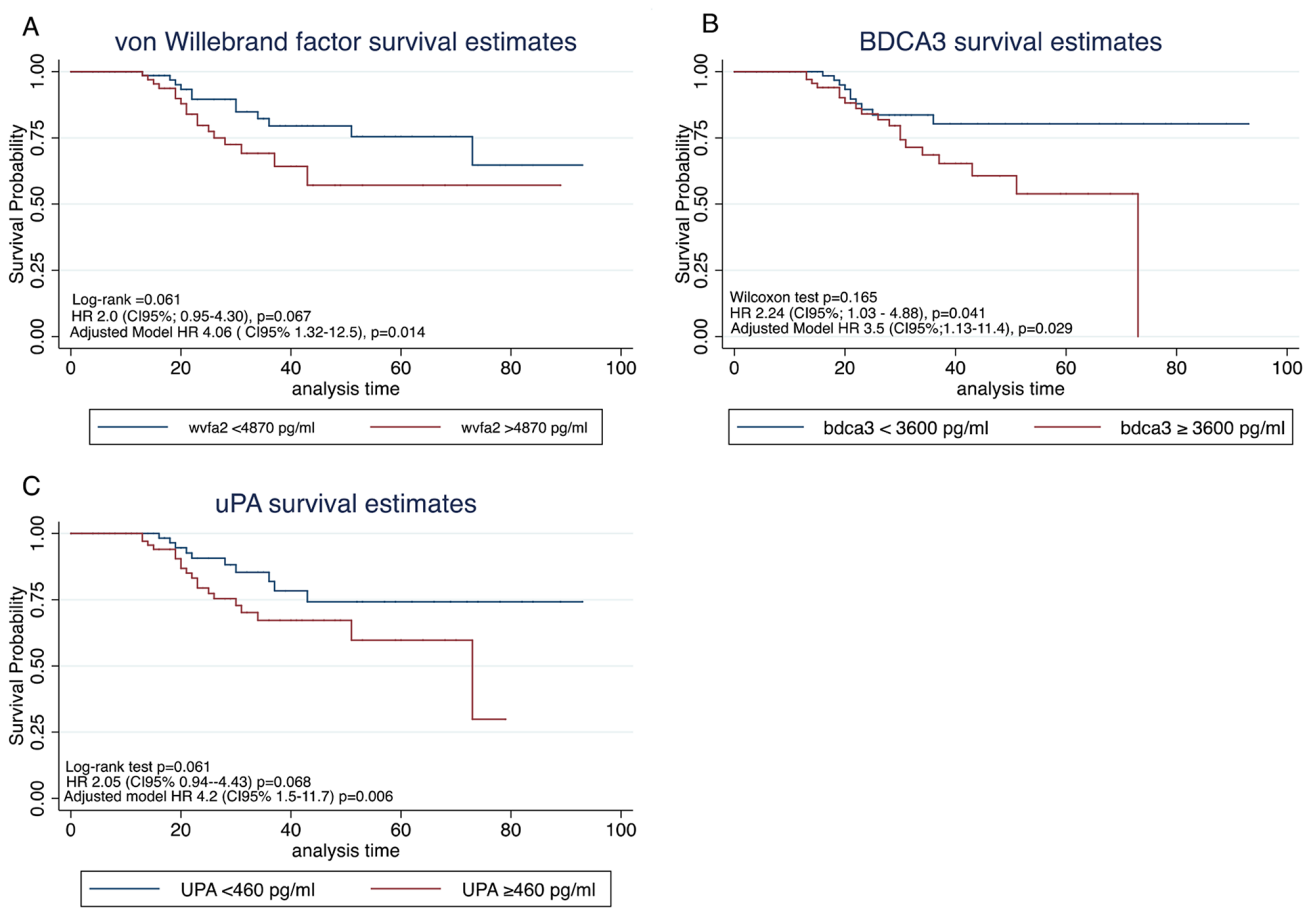
Kaplan Meier survival curves for endothelial biomarkers. (1 -**A**) von Willebrand factor analysis. (1-**B**) BDCA3 or thrombomodulin analysis. (1-**C**) uPA or Urokinase analysis. Adjusted model was done for each endothelial factor

When we evaluated the mortality rate according to VWF, those subjects with VWF under 4869 pg/ml had a mortality rate of 4.55 per 1000 person-days (95% CI: 2.58–8.01), and those with elevated VWF levels (≥ 4870 pg/ml) had a mortality rate of 8.5 per 1000 person-days (95% CI: 5.31–13.76). The biomarkers for endothelial function were tested, and the levels of each were greater in those subjects who died (Table 1). For the survival evaluation of patients according to each endothelial marker, a Kaplan-Meier curve was generated, and the hazard ratio is presented in Fig. 1A–C.

A multivariate proportional Cox model was used to evaluate the risk of death according to high levels of VWF (≥ 4870 pg/ml), BDCA3 (≥ 3600 pg/ml), and uPA (< 460 pg/ml). All variables with *p* < 0.10 in the bivariate analysis were considered for each model. Patients with elevated VWF levels had a hazard ratio (HR) of 4.06 (95% CI: 1.32–12.5, *p* = 0.014) compared to those with lower VWF levels. Additionally, patients with BDCA3 levels ≥ 3600 pg/ml had an unadjusted HR of 2.24 (95% CI: 1.03–4.88, *p* = 0.041) compared to those with lower BDCA3 levels. When adjusting for confounded variables, the HR increased to 3.5 (95% CI: 1.13–11.4, *p* = 0.029), as shown in Table 2.

**Table 2:** Multivariable proportional Cox model

Variable	Crude model		Adjusted model 1 p = 0.002		Adjusted model 2 P = 0.005	
	HR (CI95%)	*p*	HR (CI95%)	*p*	HR (CI95%)	*p*
VWF (pg/ml)*	2.02 (0.95–4.30)	0.067	4.06 (1.32–12.5)	0.014	2.58 (0.93–7.1)	0.068
BMI (kg/m2)	0.96 (0.90–1.02)	0.216	-	-	-	-
Diabetes	1.33 (0.63–2.81)	0.443	-	-	-	-
CVD	14.2 (1.7–113.8)	0.012	10.14(2.6-355.1)	0.007	12.48(0.97–160)	0.052
Nephropathy	2.74 (0.64–11.70)	0.173	-	-	-	-
Enoxaparin doses	0.97 (0.95–0.99)	0.048	0.96 (0.93–0.99)	0.017	0.97(0.97-1.00)	0.056
Previous anticoagulation	14.10 (1.76-112.84)	0.013	-	-	-	-
DHL	1.00 (0.99-1.00)	0.065	1.00 (1.00–1.00)	0.025	1.00 (0.99-1.00)	0.137
CPR	1.05 (1.00-1.09)	0.018	-	-	-	-
Procalcitonin	1.02 (0.99–1.04)	0.079	-	-	1.05(0.96–1.14)	0.235
Total Bilirubin	1.36 (1.15–1.62)	< 0.001	-	-	1.28(0.95–1.74)	0.101
IB (mg/dl)	1.22 (0.93–1.60)	0.138	0.48 (0.02–9.82)	0.639	-	-
DB (mg/dl)	1.61 (1.25–2.09)	< 0.001	2.42 (0.57–10.2)	0.175	-	-
DHL	1.00 (0.99-1.00)	0.065	-	-	-	-
Age	1.02 (0.99–1.05)	0.186	0.99 (0.95–1.03)	0.737	0.98(0.93–1.04)	0.611

DB: Direct Bilirubin; IB: indirect Bilirubin; CVD: Cerebrovascular Disease; CPR: C-reactive Protein; DHL: lactate dehydrogenase; *VWFa2 > 4870 pg/ml

## Discussion

We found that the risk of death increased fourfold in subjects with higher VWF concentrations (≥ 4870 pg/ml) compared to those with lower VWF concentrations (HR 4.06, 95% CI: 1.32–12.5, *p* = 0.014). The principal entry site of SARS-CoV-2 is through the angiotensin-converting receptor II site in the respiratory tract, but the virus moves rapidly to the vascular system with alterations in the coagulation system [9, 21, 22].

VWF is a plasma glycoprotein that captures platelets that circulate to the site of vascular injury, and subsequently mediates platelet activation and aggregation. It is an important mediator of hemostasis [23, 24]. Neutrophil extracellular traps (NETs) are the main strategy used by neutrophils to kill and clear invading microorganisms, thus protecting the host from infection. However, uncontrolled NET formation may activate inflammatory cells and cause tissue damage. VWF and NETs play key roles in the formation of thrombi in the venous and arterial systems as well as the formation of cancer-associated thrombosis [7, 25–27]. Moreover, the activation of ECs via vascular hemostasis due to the expression of CD19, nitric oxide (NO) and prostacyclin, which have anti-inflammatory and antithrombotic effects, upregulates VWF expression. Similarly, the expression of adhesion molecules such as intracellular adhesion molecule (ICAM), vascular adhesion molecule (VCAM), P selectin, E selectin, and BDCA3 promotes the adhesion of leukocytes and platelets. All these processes result in excess thrombin generation, consequently leading to fibrin clots and increasing thrombotic events [11, 28].

Moreover, complement activation leads to potentiation of this mechanism by increasing monocyte and endothelial tissue factor levels, increasing platelet activation and amplifying endothelial inflammation. This response to SARS-CoV-2 leads to hypercoagulability and immunothrombosis.

In 152 patients with COVID-19, Marco et al. [29] reported elevated VWF levels. Furthermore, patients in the intensive care unit (ICU) had higher levels of VWF than non-ICU patients. They also evaluated endothelial injury in survivors (*n* = 143) and non-survivors (*n* = 9). Non-survivors had increased levels of the VWF antigen (370.2%, 95% CI: 312–400%,*p* = 0.05) compared to survivors (143.5%, 95% CI: 104.5–218.9%, *p* < 0.05), similar to our results [29]. Likewise, Vineeth et al. [30] reported that reticuloendothelial activation markers (VWF, D-dimer, ferritin, and sCD163) were independently correlated with COVID-19 severity, with VWF (units/dl) values of 202 (95% CI: 131–253) vs. 293.5 (95% CI: 231.4–355.2) vs. 328.8 (95% CI: 272.9–384) vs. 340 (95% CI: 297–389, *p* < 0.001, for mild, moderate, severe, and critical disease, respectively. This finding suggested that the greater the VWF level is, the more severe the disease. Among the patients in the cohort, 87.4% had severe disease and mechanical ventilation at onset; the median SO_2_ concentration in the emergency department was 64.9 ± 18.2%, and more than 50% of patients had moderate (48.8%) or severe (28.8%) acute respiratory syndrome. Marco et al. [29] reported similar results among their patients. They evaluated VWF as a percentage of activity and showed a greater increase in VWF antigen in non-survivors [370.2% (312–400)] than in survivors [143.5% (104.5–218.9%)] .

Another endothelial marker in this study was BDCA3, which is a cell surface-expressed transmembrane glycoprotein also known as CD141 or thrombomodulin. The elevation of this marker suggested endothelial cell activation and dysfunction [31, 32]. Endothelial damage and a procoagulant state in COVID-19 patients have been proposed by several researchers [7, 8, 32, 33]. The current study revealed that higher BDCA3 levels (≥ 3600 pg/ml) are associated with greater mortality (Fig. 1) than lower BDCA3 levels. The risk of death was 3.5 times greater in patients with a higher BDCA3 level (HR 3.5, 95% CI: 1.13–11.4, *p* = 0.029) than in patients with a level below 3600 pg/ml. Similar results were demonstrated by Goshua et al. [34] Their ICU patients had a median of BDCA3 of 4.2 (2.6–6.5) ng/ml, while non-ICU patients had a median of 3.0 (2.6–3.2) ng/ml (*p* = 0.23). Although the results did not reach statistical significance, when patients with high soluble thrombomodulin (BDCA3) concentrations (> 3.26 ng/ml) were evaluated via Kaplan-Meier survival curves, the survival probability decreased compared to that of patients with BDCA3 concentrations less than 3.26 ng/ml. Additionally, in-hospital mortality was significantly lower among patients treated with lower concentrations of thrombomodulin than among those treated with higher concentrations (HR 5.9, 95% CI: 1.9–18.4), and among ICU patients (HR 4.5, 95% CI 1.5–14.0%). This finding was similar to our Kaplan-Meier survival curve, with a cutoff point of 3600 pg/ml [34].

Further, Vicelli Dalla Sega et al. [35] presented similar data to our results, with a total of 54 patients, 30% of whom died because of COVID-19. They measured endothelin-1, endoglin, sE-selectin, thrombomodulin, soluble vascular cell adhesion molecule 1 (sVCAM-1), and VWF at three follow-ups. They demonstrated that sVCAM-1 was a predictor of mortality; nonetheless, the other biomarkers at any time period were higher in non-survivors than in survivors. Similar results were found in our study. However, we found that BDCA3, VWF, and uPA were predictive of mortality, possibly because of our sample size, which was threefold larger than theirs. Both sets of data demonstrate that patients with COVID-19 have increased levels of endothelial biomarkers, confirming endothelial dysfunction in this disease [35].

Furthermore, in the review by Zhen W Mei et al. [36], multiple ways in which VWF acts were discussed, confirming the important role of coagulopathy caused by the SARS-CoV2 virus. They found an increased level of VWF in each phase of the disease, especially during microthrombus formation, which can indicate coagulopathy. Although they did not review the role of VWF in mortality, we confirmed that VWF, BDCA3, and uPA play important roles in systemic inflammation and severity, which can lead to increased mortality in COVID-19 patients. Additionally, knowing the contribution of the imbalance that VWF plays could impact severity and mortality with the implementation of specific therapies [36].

Concerning uPA analysis, uPA is known to be an important biomarker in the physiology and pathophysiology of COVID-19, and it acts in local fibrinolysis, inflammation, tissue repair, matrix reconstruction, and angiogenesis. It has been shown to be more prevalent in acute respiratory diseases such as systemic inflammatory response syndrome (SIRS) [37]. Our data revealed elevated levels of uPA in non-survivors and an adjusted HR of 4.2 (95% CI: 1.5–11.7, *p* = 0.006) for mortality. In contrast, Yatsenko Tetiana et al. [38] reported decreased levels of uPA in patients with COVID-19. These differences may be due to the severity of the disease and sample size. Our sample shows other severe parameters of the disease, such as, fibrinogen, C-reactive protein (CRP), Troponin, etc. Likewise, almost 90% of the sample needed intubation at onset, demonstrating greater severity of the disease. Meanwhile, the sample from Yatsenko et al. shows parameters beyond the median of our population, which can indicate the phases of COVID-19. While they have uncomplicated and complicated COVID-19 cases, ours were all severely affected, so as to further draw a distinction between the two groups of patients [38, 39].

Despite our relatively small sample size, the increase in risk determined using endothelial markers was considerable, suggesting the important impact of endothelial dysfunction, the coagulation pathway, and complement activation on COVID-19 prognosis and survival. We explored whether an improvement in endothelial function reduces COVID-19 mortality or complications, or whether endothelial function markers could be used to identify other possible treatments.

## Conclusion

The results of this study confirmed that the hypercoagulability, hyperinflammatory state, and endothelial dysfunction caused by SARS-CoV-2 may lead to vascular events in COVID-19 patients. Moreover, the more severe the COVID-19 infection is, the greater the degree of endothelial damage (VWF, BDCA3, and uPA) and the greater the association with an increased risk of mortality. The data and studies discussed in the paper demonstrate the impact of endothelial function through endothelial biomarkers. However, there is a further need to explore specific therapies for endothelial function in COVID-19 so as to mitigate the progression of the disease.

### Electronic supplementary material

Below is the link to the electronic supplementary material.

Supplementary Material 1

## Data Availability

Data used in this research are available by request. contact rnsanchezs@gmail.com.

## Abbreviations

BDCA3: Blood dendritic cell antigen-3
COVID 19: Coronavirus Disease 2019
DIC.: Disseminated intravascular coagulation
ECs.: Endothelial cells
ELISA: Human Enzyme linked Immunosorbent Assay
ICU: Intensive care unit
ENT: Extracellular Neutrophil Trap
SAH: Systemic arterial hypertension
SARS-CoV-2: Severe acute respiratory syndrome coronavirus 2
TNF-α: Tumor Necrosis Factor α
uPA: Urokinase PA
VEGF: Release Vascular Endothelial Growth Factor
VWF: Von Willebrand factor
