# Supplementary material for: Endothelial biomarkers (Von willebrand factor, BDCA3, urokinase) as predictors of mortality in COVID-19 patients: cohort study
Source: BMC Pulm Med. 2024 Jul 4;24:325. doi: 10.1186/s12890-024-03136-0 (PMC11229487; doi:10.1186/s12890-024-03136-0)
Supplement: Supplementary file 1 — Supplementary Material 1 [file 12890_2024_3136_MOESM1_ESM.docx]

**Supplementary material**

Characteristics of symptoms, signs and previous treatment of COVID-19 disease measured in the subject

| **Table S1. COVID-19 Characteristics** | | | | |
| --- | --- | --- | --- | --- |
| Variable | All  (n=165) | Survivors  (n=135) | Non-survivors  (n=30) | *p* |
| Intubation at onset  (%) (n=151) | 132 (87.4) | 101 (84.2) | 31 (100) | 0.014 |
| Days from symptom onset to hospitalization (n=129) | 9.9 ± 5.7 | 9.6 ± 4.4 | 11.1 ± 9.2 | 0.376 |
| Days of intrahospital stay (n=109) | 30.5 ±18.2 | 31.6 ± 19.1 | 26.6 ± 13.7 | 0.228 |
| Intubation days (n=90) | 24.9 ± 14.5 | 25.5 ± 15.7 | 23.2 ± 14.5 | 0.411 |
| S0_2_ in Emergency | 64.9 ± 18.2 | 65.9 ± 18.7 | 60.7 ± 15.6 | 0.149 |
| **Previous Treatment (%)** | | | | |
| Azithromycin | 38 (36.5) | 31 (38.3) | 7 (30.4) | 0.491 |
| Ceftriaxone | 26 (25) | 22 (27.2) | 4 (17.4) | 0.422 |
| Other antibiotics | 46 (43.8) | 36 (43.9) | 10 (43.5) | 0.971 |
| Ivermectin | 23 (22.1) | 18 (22.2) | 5 (21.7) | 0.961 |
| Antiviral | 15 (13.6) | 12 (13.8) | 3 (13) | 1.00 |
| Dexamethasone | 20 (19.2) | 17 (21) | 3 (13) | 0.553 |
| Paracetamol | 54 (51.4) | 42 (51.2) | 12 (52.2) | 0.935 |
| Antiviral + other antibiotics | 4 (8.9) | 2 (5.7) | 2 (20) | 0.209 |
| Azithromycin + antiviral | 7 (18.4) | 7 (22.6) | 0 | 0.309 |
| SIRA classification (%)  1  2  3 | 18 (22.5)  39 (48.8)  23 (28.8) | 17 (28.8)  30 (50.9)  12 (20.3) | 1 (4.8)  9 (42.9)  11 (52.4) | 0.009 |
| Enoxaparine doses (mg) | 50.4 ± 20.4 | 51.3 ± 21 | 47.2 ± 18.1 | 0.378 |
| **Symptoms of disease (%)** | | | | |
| Dyspnea | 139 (96.5) | 110 (96.5) | 29 (96.7) | 1 |
| Cough | 93 (65.5) | 76 (67.3) | 17 (58.6) | 0.383 |
| Thoracic pain | 20 (14.1) | 17 (15.0) | 3 (10.3) | 0.765 |
| Fatigue | 81 (57.0) | 65 (57.5) | 16 (55.2) | 0.820 |
| Sputum | 19 (13.4) | 17 (15) | 2 (6.9) | 0.364 |
| Fever | 113 (80.1) | 86 (75.5) | 27 (90) | 0.196 |
| Odynophagia | 56 (39.4) | 43 (38.1) | 13 (44.8) | 0.505 |
| Headache | 60 (42.3) | 45 (39.8) | 15 (51.7) | 0.247 |
| Diarrhea | 16 (11.3) | 13 (11.5) | 3 (10.3) | 1 |
| Conjunctivitis | 2 (1.4) | 1 (0.9) | 1 (3.5) | 0.368 |
| Anosmia | 6 (4.3) | 6 (5.4) | 0 | 0.346 |
| Ageusia | 3 (2.1) | 2 (2.7) | 0 | 1 |
| Myalgias | 107 (75.4) | 83 (73.5) | 24 (82.8) | 0.345 |
| Arthralgias | 94 (66.2) | 71 (62.8) | 23 (79.3) | 0.094 |
| Chi square or F fisher test was done for ~~v.~~ qualitative and independent variables; Student’s t or Mann-Whitney U for quantitative variables. | | | | |
